# A hybrid computational approach to anticipate individuals in sequential problem solving

**DOI:** 10.3389/frai.2023.1223251

**Published:** 2023-12-18

**Authors:** Giacomo Zamprogno, Emmanuelle Dietz, Linda Heimisch, Nele Russwinkel

**Affiliations:** ^1^Department of Computer Science and Engineering, University of Bologna, Bologna, Italy; ^2^Airbus Central Research and Technology, Hamburg, Germany; ^3^Department of Psychology and Ergonomics, Technische Universität Berlin, Berlin, Germany; ^4^Group Human-Aware AI, Institut of information Systems, Universität zu Lübeck, Lübeck, Germany

**Keywords:** cognitive modeling, hybrid (symbolic sub-symbolic) approach, sequential problem solving, anticipation, human-robot collaboration (HRC)

## Abstract

Human-awareness is an ever more important requirement for AI systems that are designed to assist humans with daily physical interactions and problem solving. This is especially true for patients that need support to stay as independent as possible. To be human-aware, an AI should be able to anticipate the intentions of the individual humans it interacts with, in order to understand the difficulties and limitations they are facing and to adapt accordingly. While data-driven AI approaches have recently gained a lot of attention, more research is needed on assistive AI systems that can develop models of their partners' goals to offer proactive support without needing a lot of training trials for new problems. We propose an integrated AI system that can anticipate actions of individual humans to contribute to the foundations of trustworthy human-robot interaction. We test this in Tangram, which is an exemplary sequential problem solving task that requires dynamic decision making. In this task the sequences of steps to the goal might be variable and not known by the system. These are aspects that are also recognized as real world challenges for robotic systems. A hybrid approach based on the cognitive architecture ACT-R is presented that is not purely data-driven but includes cognitive principles, meaning heuristics that guide human decisions. Core of this Cognitive Tangram Solver (CTS) framework is an ACT-R cognitive model that simulates human problem solving behavior in action, recognizes possible dead ends and identifies ways forward. Based on this model, the CTS anticipates and adapts its predictions about the next action to take in any given situation. We executed an empirical study and collected data from 40 participants. The predictions made by CTS were evaluated with the participants' behavior, including comparative statistics as well as prediction accuracy. The model's anticipations compared to the human test data provide support for justifying further steps built upon our conceptual approach.

## 1 Introduction

The ability to adapt to a developing situation is a highly desired feature in every-day interaction. Humans are incredibly good at adapting to their environment and in interaction with other humans or (artificial) systems. This skill enables humans to enhance their performance and the performance of others in the environment.

In recent years, various disciplines that aim at implementing AI systems and robotic systems within an interactive environment have realized the need for human-aware AI or human-centered AI design (Kambhampati, 2020). Kambhampati (2020) defined Human-Aware AI Systems as goal-directed autonomous systems that are capable of effectively interacting, collaborating, and teaming with humans. Challenges in designing such human-aware AI systems include modeling the mental states of humans-in-the-loop, recognizing their desires and intentions, providing proactive support, exhibiting explicable behavior, giving cogent explanations on demand, and engendering trust.

While recent advances have focused on the emotional aspect of interaction, successful human-robot interaction requires the machine to understand, and model the intentions and strategies of the individual humans they interact with in order to adapt to the human partner (Rossi et al., 2017). It has been shown that interactions with robotic systems that do not show adequate feedback will cause frustration for their human cooperation partners (Weidemann and Russwinkel, 2021).

This is especially important for patients suffering from Parkinson's disease or other neural impairments that face challenges living on their own or being dependent on others even for simple tasks. 20% of the world's population lives with some level of cognitive impairment (World Health Organization and others, 2011). According to Kosch et al. (2018) an individual with such impairments may have difficulties in learning, remembering information, or making decisions. A cognitive impairment can impact someone's ability to complete traditional activities of daily living. Different facilities exist to provide specialized training to people with cognitive impairments as they learn independent living skills. The ultimative goal is to teach the patients to live independently. Contextualized assistance has been shown to be effective in helping people with cognitive impairments perform individual work. So far only displays or augmented reality tools have been used but with cognitive anticipative models future systems could provide better support. An assistance system that is adaptive to the individual and the task progress, that would understand the task state and offer adaptive support could ensure independence of such patients to a crucial degree. It has been shown that self efficiency for such tasks is crucial to preserve still intact skills and self esteem. But assistance systems that adapt to situation, task and context would enable a new generation of human-aware AI systems. We believe that the ability to anticipate the intentions and cognitive processes of a person is a key skill that underlies their adaptability. Especially the ability to anticipate high level (taks level) and low level intentions (actions) (Gomez Cubero and Rehm, 2021) is crucial to proactively offer support or anticipate dangerous situations in time.

According to Klein et al. (2007) anticipatory thinking is a critical macrocognitive function of individuals and teams. It is the ability to prepare in time for problems and opportunities. We distinguish it from prediction because anticipatory thinking is functional–people are preparing themselves for future events, not simply predicting what might happen. Therefore anticipation implies the skill to understand the task structure the partner is in and difficulties the partner is facing. For assisting the partner in a task where the final solution might not be known but - as a teacher - the assistant can still be aware of general pitfalls and short term solutions for the next step.

First steps toward such a goal would be an approach that

- is able to offer support even if the final goal is not known (or trained) in detail,
- understands state of the task and difficulties related to it,
- understands common mistakes and the problem that the individual partner might face,
- anticipates the partner,
- is flexible to the individual trace of actions, and
- offers appropriate support and only when needed, without taking over from the patient or partner.

Taking these requirements as a starting point, we will present an integrated AI system that has a computational theory of mind, which it uses to anticipate actions of individuals for a particular task and adapt according to previous anticipations. In real world tasks we are often faced with developing situations - each action or decision changes the state of the problem which is referred to as dynamic decision making.

According to Brehmer (1992) dynamic decision making occurs under conditions which require a series of decisions, where the decisions are not independent, where the state of the world changes, both autonomously and as a consequence of the decision maker's actions, and where the decisions have to be made in real time.

As a first step we developed an approach that involves dynamic decision making, and is simple to understand and investigate, but is sufficiently complex having a broad solution space where each step changes the problem state. The sequential problem solving task *Tangram* fits this requirement. This task will be explained in more detail in Section 3. Whenever a piece is chosen and placed the remaining pieces and remaining free spaces change - so we have a new situation. A solution with machine learning would need a lot of training trials for each problem and does not seem appropriate. On the other hand, when observing other humans solving the task, it seems straightforward for a human to understand the other's perspective and recognize their intent for the next move.

Regarding the requirement above of anticipating the partner a method is needed for this task that can trace the individual action decisions of a participant and apply general rules to understand the situation and possible approaching problems in order to support but not necessarily taking over the task.

Especially for patient-robot collaboration human-aware capabilities like non-verbal communication, shared-control and proactive support are crucial for successful patient assistance and therefore require new solutions how to take into account the individual in the task. In addition cognitive principles should be taken into account which are hypotheses for human strategies, one such cognitive principle can be affordance. According to Gibson (2014), the use of an object is intrinsically determined by its physical shape. Such clues indicate possibilities for action in a task. They are perceived in a direct, immediate way and might also lead to wrong solutions as we will show. In order to anticipate a person in a task it is necessary to include such cognitive principles to understand intentions and errors.

Different than purely data-driven approaches, we will present a hybrid approach, which exploits the strengths of various technologies. The paper's contribution is three-fold:

(a) The results and analysis of an empirical study to a problem solving geometrical task (the Tangram) that, while conceptually simple, requires skills that can probably be generalized to more complex tasks.
(b) A novel hypothesis for human strategies, so-called cognitive principles, for the case of the sequential problem solving task is suggested, taking as a baseline the concept of affordance and providing different variations thereof.
(c) A computational framework integrating the techniques of object recognition and modeling with cognitive architectures to enable an agent to reproduce the strategies and provides basic insights toward predicting and/or anticipating human behavior in the solution process. This system shows how the strengths of machine learning and the inherently explainable cognitive modeling approach can be combined. Finally, four different evaluation methods are presented to evaluate the framework, which, while extending on the main plan of studying the underlying principles, we hope will provide a basic benchmark for future studies.

## 2 Related work

### 2.1 Macro-cognition

The concept of macrocognition is a way of describing cognitive work as it naturally occurs (Klein and Wright, 2016). It is a term to indicate a level of description of the cognitive functions that are performed in natural decision-making settings. According to Klein and Wright (2016) important macrocognitive phenomena are problem detection, situation assessment, attention management and uncertainty management. The term comprises the mental activities that must be successfully accomplished to perform a task or achieve a goal. And this is needed for a human-aware AI approach that understands the partners task state on such a macrocognitive level. It is relevant to have tools that can achieve “understanding” at this macrocognitive level for supporting a human in a difficult task. This is sense making at an abstract task or situation understanding. Understanding that a person got herself in a dead-end or tries to find an impossible solution can provide valuable support. Not for every situation there will be a solution available but a macrocognitive understanding of a problem is high relevant.

### 2.2 Human-aware approaches

During the last decades, theories on human reasoning that aimed to understand, model, and eventually predict their decisions [e.g. Johnson-Laird (1983), Rips (1994), Polk and Newell (1995), Chater and Oaksford (1999), Oaksford and Chater (2020), and Knauff and Gazzo Castañeda (2021)] can be seen as a theoretical foundation on human-awareness. These approaches differ fundamentally from the normative requirements of classical logical reasoning. The current two most dominant paradigms are either based on the mental model theory (Knauff and Gazzo Castañeda, 2021) or bayesian (Oaksford and Chater, 2020). Another view is to formalize commonly agreed-to patterns as cognitive principles and parametrize them to account for the variety among the human population [e.g., Dietz Saldanha and Schambach (2020)] and applied to cognitive argumentation [e.g., Dietz Saldanha and Kakas (2019)]. Dietz et al. (2022) integrated this approach for quantitative symbolic reasoning including the notion of Bayesian plausibility. Cognitive modeling in real world environments toward human-aware systems in the context of cockpit and pilots' mental state was done in (Klaproth et al., 2020; Blum et al., 2022). These approaches integrated the pilots' neurophysiological responses from a passive brain-computer interface within a cognitive model to trace pilots perception and processing of auditory alerts and messages during operations. Their work demonstrates how cognitive models can be complemented with the neurophysiological data for adaptation and action (sequence) anticipation. Another human-aware approach was implemented by Scharfe-Scherf et al. (2022) in the domain of highly automated driving. The question of how much time a driver needs to safely transition from autonomous driving to manual driving is still debated. Scharfe-Scherf et al. (2022) developed a cognitive model that simulates the construction of situation awareness depending on gazes to the relevant objects next to the car. This way the prediction of transition times depending on the complexity of the situation were possible. Dietz and Klaproth (2021) proposed the cognitive modeling library *txt2actr* which provides an interface between the environment specification and the cognitive architecture ACT-R (Anderson, 2007). It automates the construction of knowledge about the task environment and facilitates updating new information in dynamic environments, such as constantly changing values in milliseconds (Scharfe-Scherf et al., 2022).

### 2.3 Theory of mind and cognitive architectures

The capability to understand someone else's purposes, intentions and goals seems to be a task that is simple for humans but difficult for AI systems (Lieto, 2021). One reason for this capability is often explained by the theory of mind (TOM) (Premack and Woodruff, 1978) stating that usually humans (from the age of 4 years on) can imagine themselves into someone's else mental state, that is they can take another person's perspective without the actual experience. One assumption is that if we know how to build the human mind computationally, we should be able to rebuild and simulate human behavior. This simulation could help AI systems to understand others' purposes, intentions and goals and adapt accordingly. According to Newell, a theory of the human mind should address all aspects of cognition. For this purpose he suggests to develop cognitive architectures (Newell, 1990), which unify different information processing structures. ACT-R (Anderson, 2007) and SOAR (Laird, 2012) are such architectures, allowing the simulation of cognitive processes. These architectures have made a significant contribution on building a baseline for formal methodologies by implementing and evaluating models based on existing theories. However, these architectures allow a high degree of freedom. Therefore the “standard model of the mind” (or “common model of cognition”) has been proposed by Laird et al. (2017) to “facilitate shared cumulative progress” and align theories on the architectural level. When considering again the challenges of human-aware AI systems, then Cognitive architectures could help overcome challenges of human-aware AI in understanding the human's awareness of a given environment. Ideally, “knowing” the human's perspective, the AI system should be capable to adapt accordingly.

### 2.4 Problem solving tasks in education and medicine

Problem solving tasks have been repeatedly suggested and implemented in education in order to enhance learners mathematical and spatial skills (Lee et al., 2009; Judd and Klingberg, 2021). This can apply to a wide range of possible targets, from children (Bohning and Althouse, 1997) to elderly people (García et al., 2010; Frutos-Pascual et al., 2012). A cognitive model for the solution mechanism of puzzles could help support and improve already present techniques: first, it would offer a reference to compare with the results of the learner, possibly helping to target weaker points or understanding reasoning patterns and causes of mistakes. Secondly, as more and more automated systems start taking the roles of supporting agents, a system provided with some type of TOM could offer better understanding and support to the users.

This section has given a brief overview of the topics that are related to the approach we will propose in this paper: a Computational Human-Aware System for Sequential Problem Solving guided by Cognitive Principles. Social robotics and human-machine teaming require human aware AI, thus a system that has the capability to anticipate and adapt to their human users. Taking this challenge as a starting point we consider a specific sequential problem solving task (Section 3) and present a computational system that anticipates and adapts its predictions based on the simulation within a cognitive architecture (Section 5). The baseline of this system is provided by theories from macro-cognition and human-aware task analysis which are grounded in so-called cognitive principles (Section 4).

## 3 The Tangram study

### 3.1 The Tangram puzzle

The Tangram is an ancient Chinese puzzle in which seven pieces, also called *tans*, are obtained from an original square and need to be reorganized into different figures.

**Figure 1:**
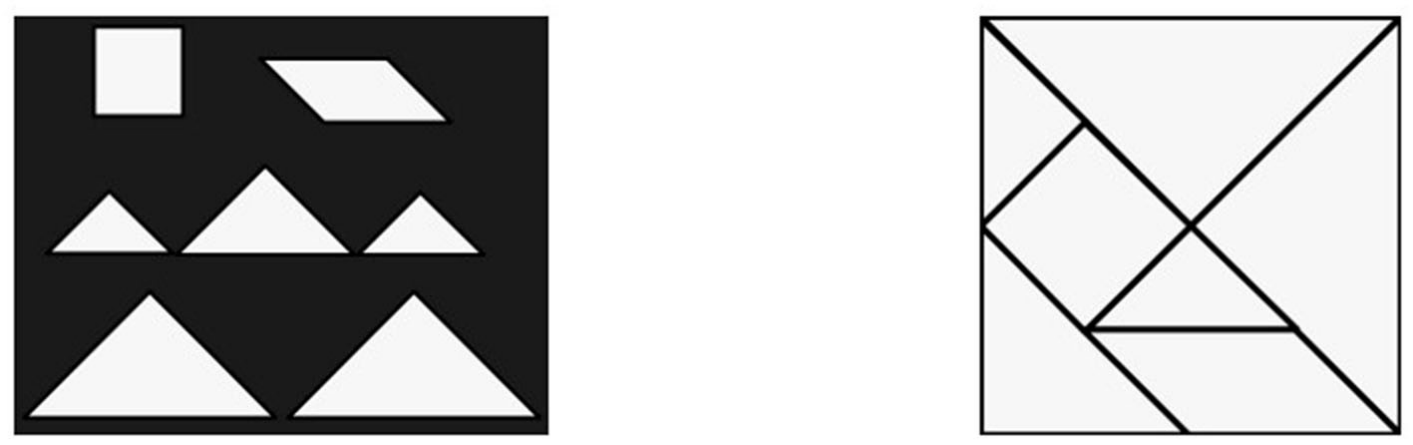
The seven tans **(left)** and their usual starting position **(right)**.

The seven tans consist of 5 square triangles (2 small, 1 medium and 2 large), 1 square and 1 parallelogram, their relative dimensions and sizes is shown in Figure 1 left.^1^

Usually players are presented with a homogeneous silhouette, likely in the shape of some stylized figure, and are asked to reproduce the pattern by positioning all the tans, without overlaps. Figure 1 right shows their usual starting position as presented to the players. Throughout the paper, we will use the symbols □, □, 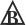, 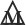, and ⧌ to refer to the square, parallelogram, big triangle, middle triangle and small triangle, respectively. The advantage of Tangram as use case for modeling cognitive solving behavior comes from two factors. First, it is relatively easy to define and describe, with a short set of rules and limited ambiguity. Second, it is an example of a sequential problem solving task, in the sense that different sequences of steps can lead to very different solution procedures, and thus it cannot be simply defined by a deterministic evolution of states.

These factors contribute in making the Tangram a solid starting point for guiding research in sequential problem solving. If a plausible TOM is suggested for the puzzle, it might provide support to the fact that a similar approach can be further applied to practical tasks that, while more complex and elaborate, share common features with the Tangram, examples of which could be educational and rehabilitation activities.

In addition, the inclusion of the Tangram puzzle in HCI for education and medicine is a topic that despite a recent rise in interest (García et al., 2010; Frutos-Pascual et al., 2012; Kirschner et al., 2016) still offers plenty of space for research effort. As the usage of Tangram as an educational tool has been known for some years, developing HCI solutions based on the puzzle seem a natural and promising advance that could provide new tools for instructors and assistants alike. Until now, computer-based assistants for Tangram like the ones mentioned above have been based on search algorithm or machine-learning solutions and a natural extension would thus be agents that model and understand their partners' solution processes, in order to better interface with users and provide more explainable support.

### 3.2 Empirical study scenario

In collaboration with the Technical University of Berlin (TUB) and Airbus Central R&T, Hamburg, an empirical study was developed and performed in order to obtain the training and test sets.

Altogether, 40 participants were acquired in two study phases, where one was dedicated to gather data for the later training and one for the later test set. Participants were recruited among students and PhD students at TU Berlin. In the first study phase, 31 participants were acquired (13 female), with a mean age of 21.53 years (*SD* = 3.21), ranging from 21 to 34 years. In the second study phase, 9 participants were acquired (6 female), with a mean age of 26.11 years (*SD* = 4.91), ranging from 20 to 35 years.

All participants signed an informed consent and were compensated with miniature aircraft models provided by the company and course credit, where applicable.

**Figure 2:**
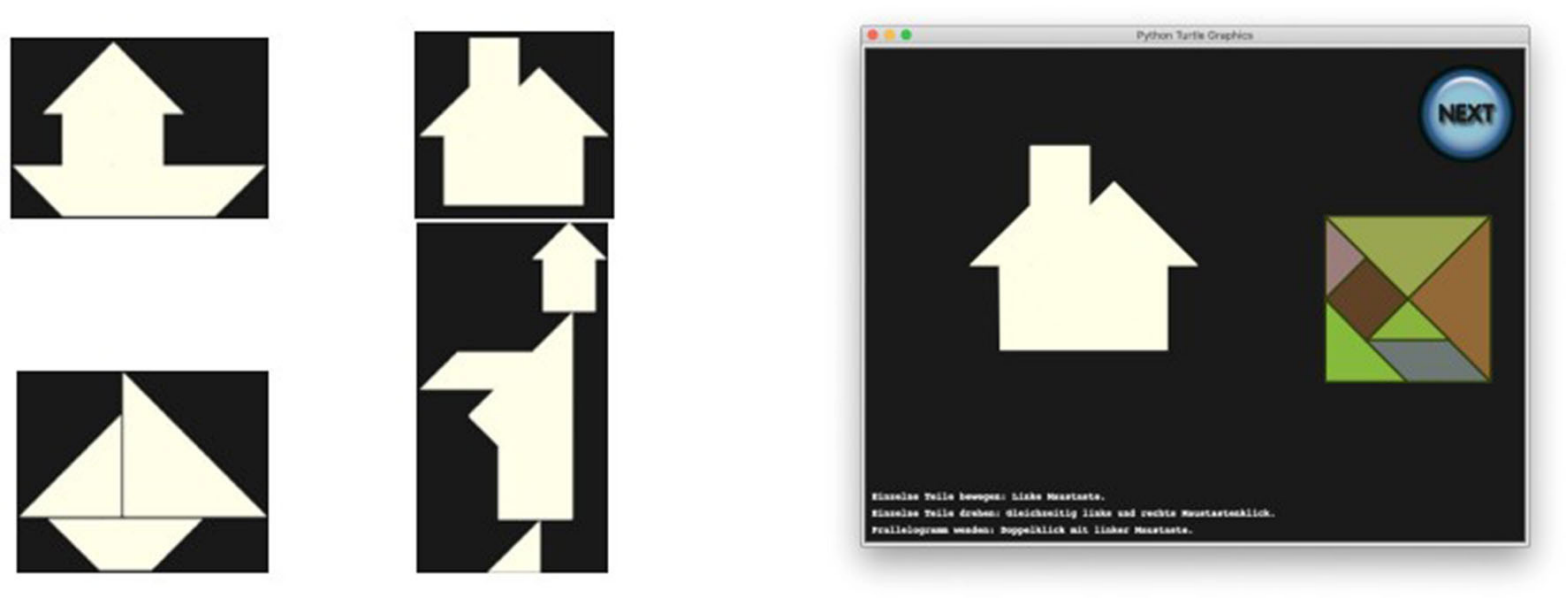
Tangrams to be solved in the empirical study were house boat, house with chimney, sailing boat and monk **(left)**. Screenshot when house with chimney was presented **(right)**.

Each participant was presented with a virtual implementation of the Tangram puzzle running on a desktop computer, consisting of one of the four puzzles on the left of Figure 2. A screenshot is shown on the right of Figure 2. After an initial explanation of the controls, all of which were performed with the mouse, each participant was required to tackle the problem of the puzzles, which were always shown in the same order. There was no time limit, and at any moment a *NEXT* button was available, so that the player could give up on the specific Tangram and move to the next one. While backtracking was possible while working on a single task, once the button was pressed the solution was submitted and no option to go back was available.

The screen recording and application logs, including data recording times, piece action types (rotation, movement) and piece positioning were stored for the analysis. For simplicity, in the sequel, we will only discuss the House and Monk.

## 4 Cognitive principles

Humans seem to make assumptions and apply a variety of heuristics that guide their decisions, which are heavily context dependent. We call the generalization of these observed heuristics *cognitive principles*. The relevant cognitive principles that address the observations made while watching the videos from the training data of the Tangram task introduced in Section 3 will be introduced in this section.

### 4.1 Affordance

In Human-Computer Interaction, immediately perceived possible actions are often referred to as affordances (Norman, 2002), and originate from Gibson (1979). The solving process for Tangrams across participants seems to be strongly guided by such immediately perceived possible actions. In particular it seems that participants first consider features in the silhouette that are particularly similar to the available piece. We call this the best fit principle.

**Figure 3:**
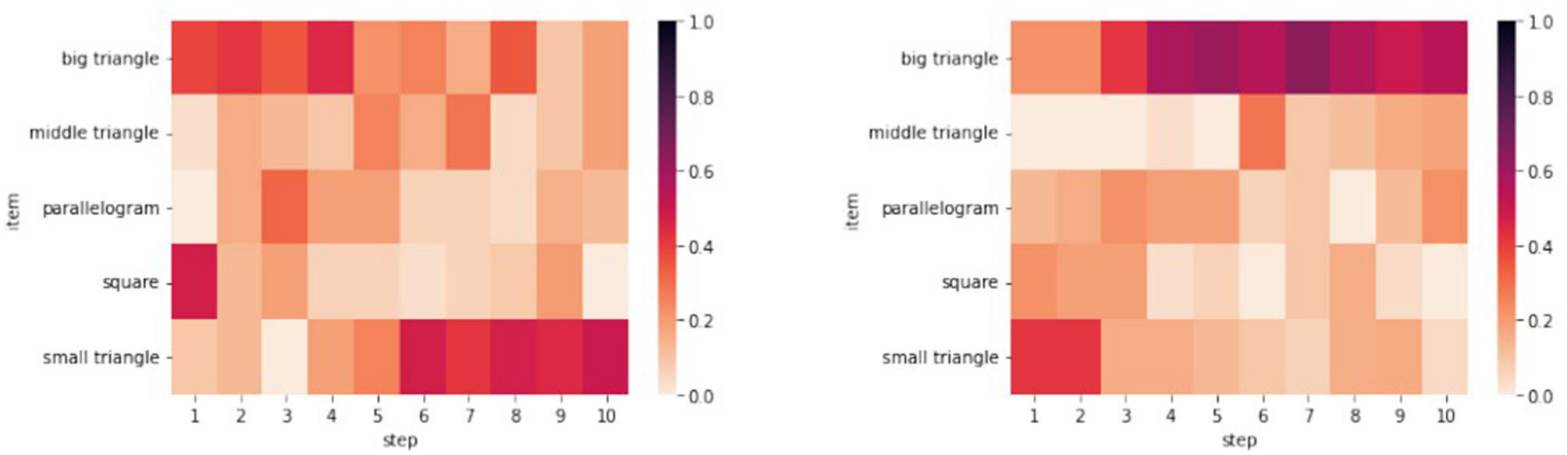
Frequency of chosen pieces by step for House **(left)** and Monk **(right)**.

**Figure 4:**
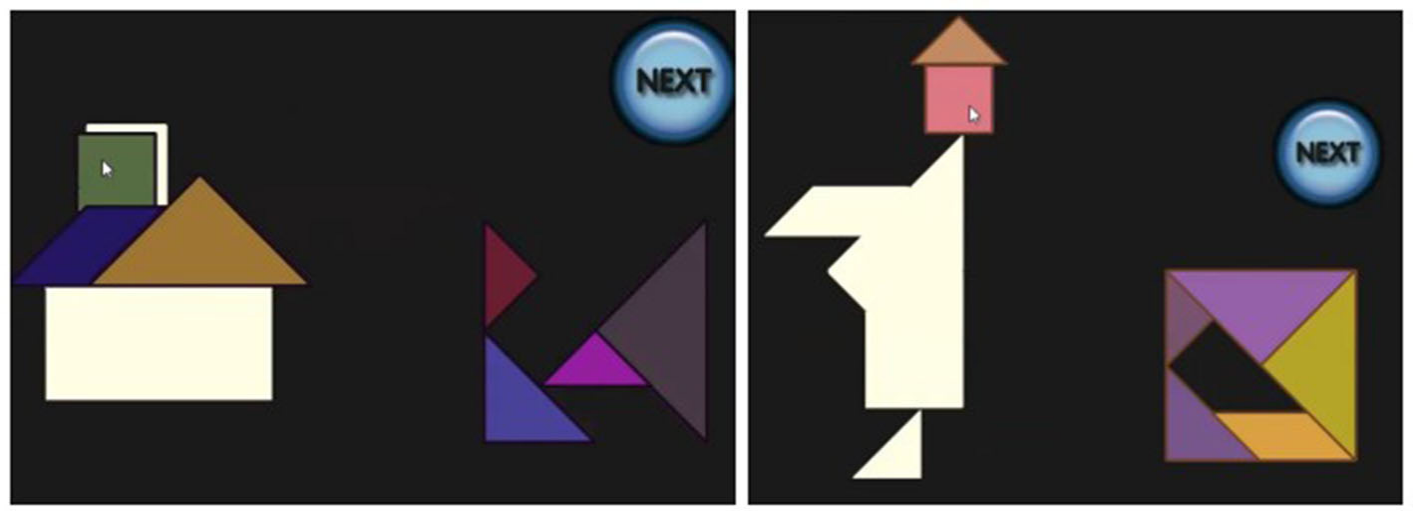
Common pattern for House **(left)** and Monk **(right)** Tangram.

Let us illustrate this observation by considering the training data from the House and the Monk Tangram: The heatmap in Figure 3 left shows that the pattern {□, 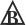, □} appears in the majority of activities in the first steps. This pattern, intended as the unordered set of chosen pieces, would compose the upper section of the house in the puzzle's solution (see Figure 4, left): the sequence with which the pieces were chosen varies, but an initial analysis shows that 47% of participants presented this pattern by step 4, rising to 77% for pattern {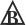, □}. Figure 3 right shows a heatmap produced by the training data for the Monk Tangram, where pattern {□, ⧌} is present in 60% of participants by step 4. This pattern would compose the head of the monk in the puzzle's solution (see Figure 4, right).

A common feature of the patterns {□, 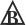, □} and {□, ⧌}, is that the shape of the area that the chosen pieces are covering clearly resembles (or coincides with) some parts of the shape of the object itself. Screen recordings showed that these combinations of actions were shared among the participants, especially during the early phases of the solution (see Section 4.4 for the phases of solving principle). While the specific sequence of steps might vary, when considering the unordered set of steps, the same patterns of action combinations can be found in the majority of the participants.

### 4.2 Initial placement errors

**Figure 5:**
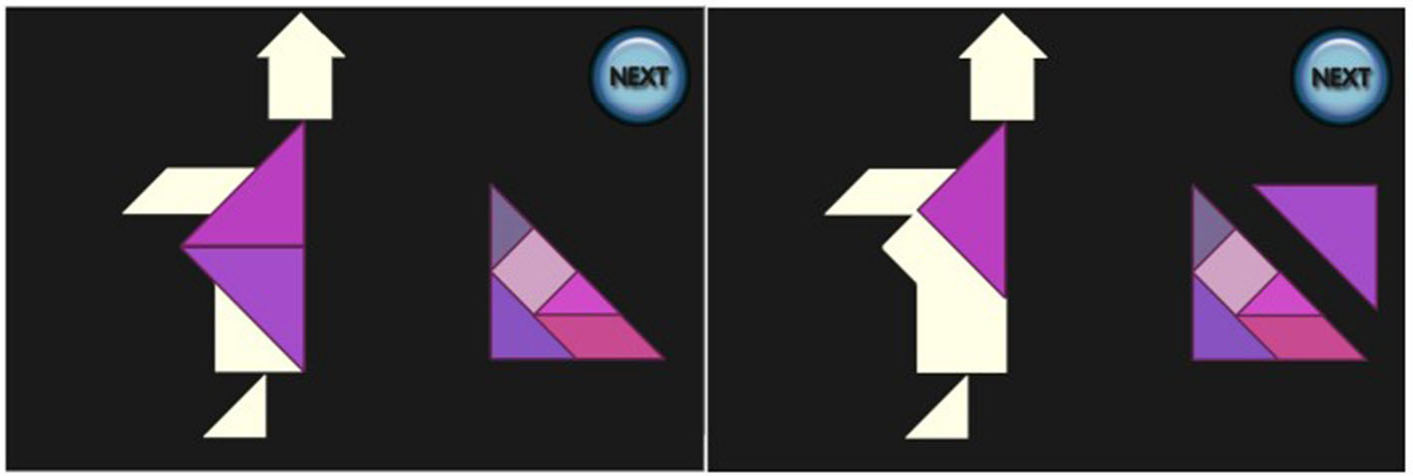
Correct placements **(left)** and wrong placement **(right)**.

Best fits which require the composition of more than one tan are not always recognized in the initial phase. Consider the placement in Figure 5 left, where the two big triangles need to be composed into a huge triangle in order to perfectly fit the “belly” of the Monk. Quite consistently participants tend to first place a big triangle rotated so to match the rotation of the larger silhouette, aligned along one of the available edges as in Figure 5 right. They do not seem to notice the possibility of a perfect fit by the arrangement of the two triangles into the bigger one.

**Figure 6:**
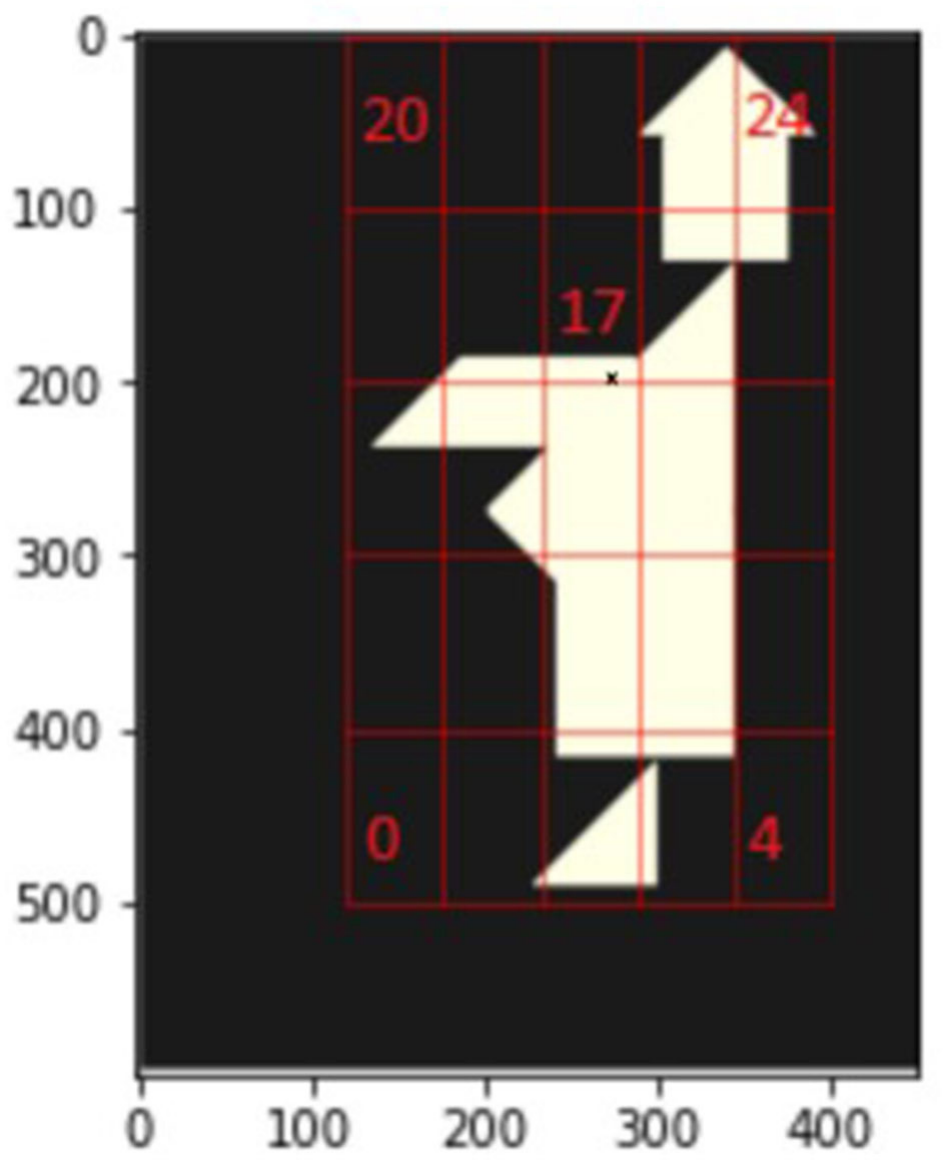
Grid layout of Monk.

This can be observed in the starting phase of the Monk Tangram: Table 1 shows the frequencies of action taken by participants in the first 4 moves and it can be noticed how 47.5% of participants' actions involve big triangles rotated 270 degrees and put at location 17 in Figure 6,^2^ representing the incorrect positioning, against a combined 20% from the correct placements. In the sequel, we will call observations of this kind the unrecognized composition principle.

**Table 1:** Action frequencies of big triangle at initial phase.

**Grid value**	**Rotation**	**Choice in %**
7	45	10
7	90	5
8	90	2.5
8	270	2.5
9	180	2.5
10	225	2.5
12	45	2.5
12	135	10
12	180	2.5
13	270	47.5
14	90	2.5
14	180	2.5
17	135	7.5

### 4.3 Backtracking

**Figure 7:**
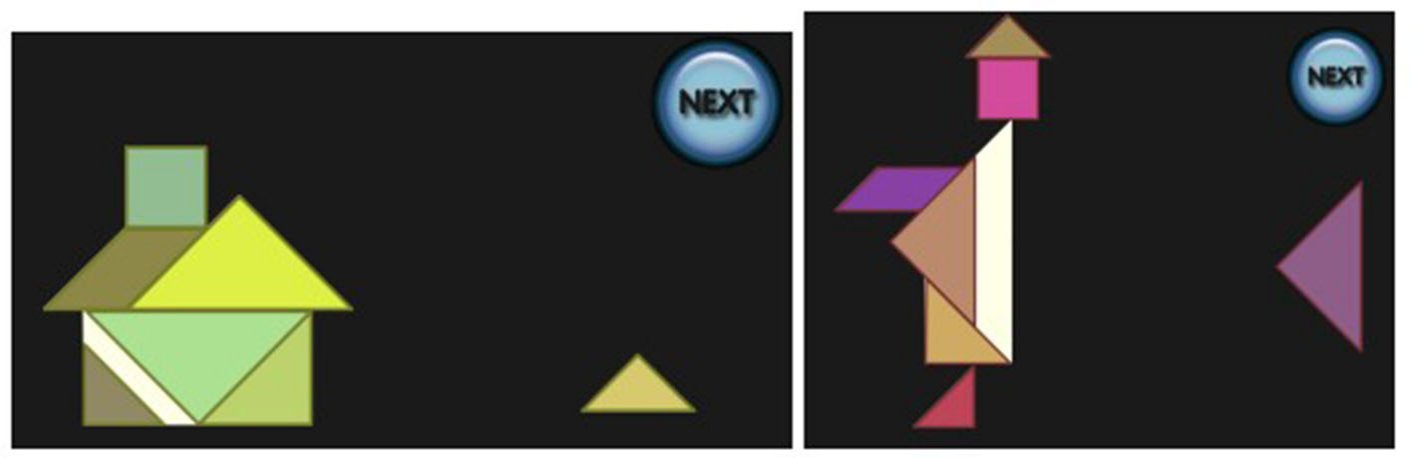
The uncovered areas in the House **(left)** and Monk **(right)** are unfeasible regions.

The concept of backtracking involves any action or strategy aimed at removing or replacing tans that have already been moved into the silhouette. In a preliminary assessment of the data, we can observe two types of backtracking. The unfeasible region backtracking occurs when a region in the silhouette is considered *unfeasible*, i.e. it clearly cannot host any of the available pieces. Noticing the presence of such region clearly hints at the unfeasibility of the current partial solution and thus triggers backtracking. See Figure 7 for two examples. This type of backtracking should involve one (or all) the pieces creating the region, i.e. the pieces bordering with it. It is important to specify that this strategy triggers when the *unfeasible region* is noticed, not produced. It can thus be the case that further action are taken after its creation, but when the participant backtrack, they would first remove the problematic piece, possibly allowing following actions. This observation is called the unfeasible region backtrack principle.

The second type of backtracking occurs even if no problematic regions are currently present. Participants might still decide to backtrack anyway. Possibly, the participants might have noticed that any further placement will trigger an unfeasible region, or that a previously placed tan did not result in a simple and clear path to the solution. In this case, there is not a clear candidate piece to be backtracked, but usually it can be expected that the piece that is not a best fit might be a possible choice for backtracking. This observation is called the piece backtrack principle.

### 4.4 Phases of solving, random search and aha moments

The aforementioned principles appear to be present at different points in the solution process. Particularly, a common trend for a player is to start by exploiting the best fit principle (see Section 4.1) until a mistake is made and an unfeasible region is found. In the analyzed Tangrams this often happens due to mistakes that can be traced back to the unrecognized composition (See Section 4.2 on initial placement errors). Backtracking follows, possibly combined with repetitions of combination mistakes, until a particular action is taken, which might trigger an aha moment [aha Moment principle, see also Schulte (2005)]. The core strategy of the second phase was described as “random search” [random search principle, see also Wilson (2002)]: lacking clearer affordances, Aha moments can be often clearly noticed by video observations, as the search phase is followed by a very quick solution obtained by best fit. Trials following these steps can thus be generally split in three phases: the starting phase, where best fit is dominant, exploration phase, showing a combination of unrecognized composition, random search possibly including piece backtrack and unfeasible region backtrack (see Section 4.3), and eventually, the final phase, mostly including aha Moment, characterized once more by best fit.

In cases where the solution is accomplished by the minimal number of necessary steps, the starting phase and the final phase are present, but the exploration phase is missing.

## 5 Cognitive Tangram Solver

We believe that the best way to understand how humans address sequential problem solving, or in particular, how they solve Tangrams, is by building a computational system which simulates their behavior. Therefore, we have developed the Cognitive Tangram Solver (CTS) framwork, whose aim is to behave as a human Tangram player. Given this objective, it was evident to implement the decision making process in a cognitive architecture. Cognitive architectures, especially in the form of hybrid architectures (combining symbolic and subsymbolic methods), can represent an interesting middle-ground approach to modeling the cognitive principles introduced in Section 4. The goal is to reproduce computationally the mechanisms of human cognition, using such mechanism as foundation and justification for the intelligent behavior of the system (Lieto et al., 2018). The theoretically founded nature of cognitive architectures imposes constraints and limits to the modeling process, but it provides also inherent explicability and plausibility properties once the system is functional. We chose the cognitive architecture ACT-R, as it provides a wide range of functionalities, is well established within the scientific community and has a very well documented manual (Bothell, 2022) including an extensive tutorial.

While solving a Tangram puzzle relies heavily on the visual perception (such as affordances, see Section 4.1) there is no such straightforward way to extract elaborate geometric shapes in an acceptable time through the visual module in ACT-R. Therefore, an additional visual-system module outside of ACT-R was developed to cover this functionality.

**Figure 8:**
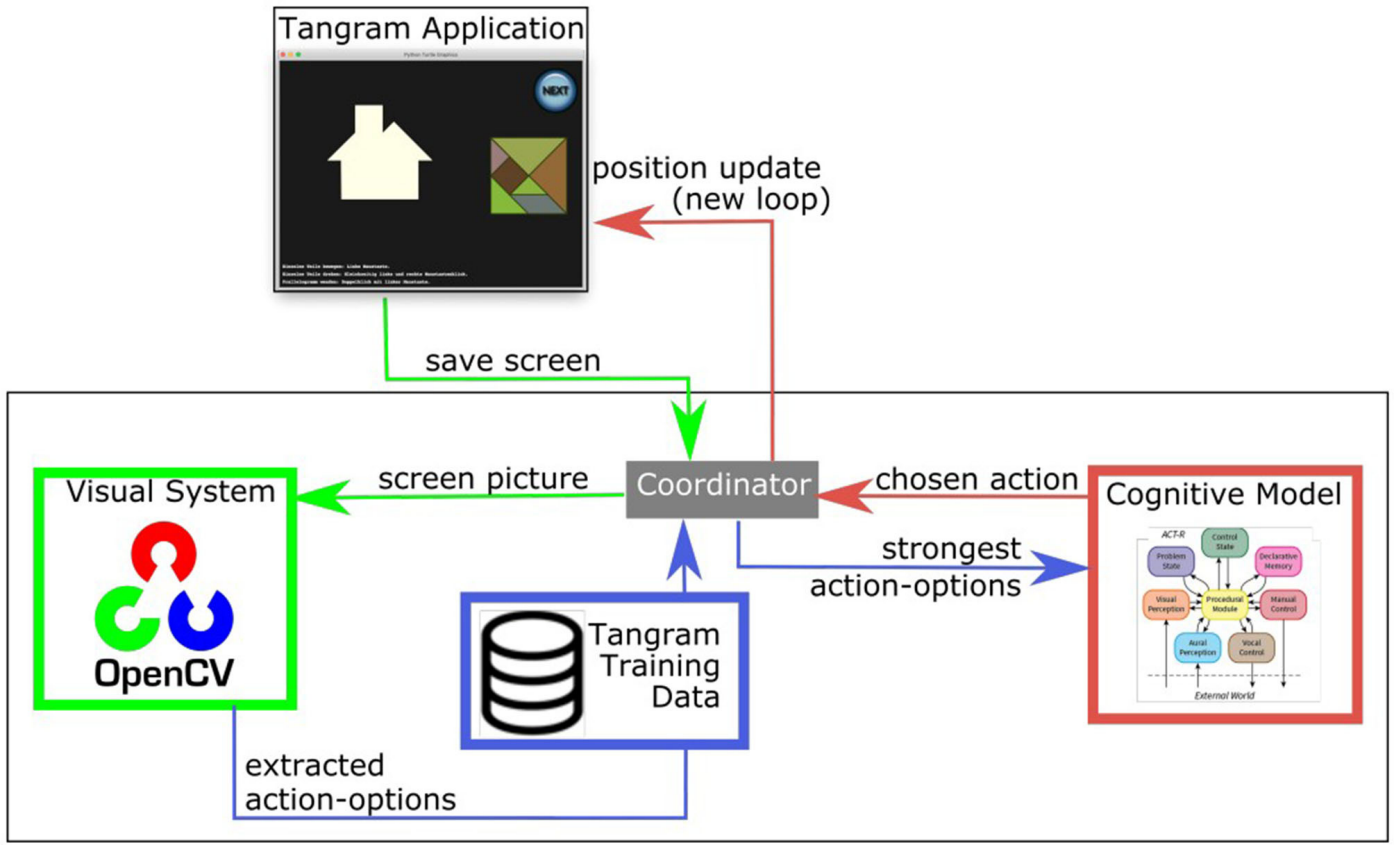
Overview of the Cognitive Tangram Solver framework.

Figure 8 gives an overview of the different modules and their functions in the Cognitive Tangram Solver framework. The coordinator module (Section 5.1) handles the interaction of multiple sub-components (Section 5.2-5.4). These sub-components are distinct and provide various functionalities: the *Tangram application* module (Section 5.2) creates and updates the empirical study window, the *visual-system* module (Section 5.3) extracts “plausible” action-options in the current state and the *cognitive model* module (Section 5.4) performs the main reasoning task based on which it selects the next action.

The implementation of the Cognitive Tangram Solving and two videos that show the application in action can be found here: https://github.com/Thezamp/tangram-solver.

### 5.1 Coordinator

The coordinator is an application written in python, which is central as it provides the interfacing features between the other modules. It initializes the various sub-components, keeps an internal representation of the puzzle state and updates it according to the action-option chosen by the cognitive model.

#### 5.1.1 ACTION-OPTIONS

An action-option is the interfacing element between the visual system, the training data and the cognitive model and based on the following assumptions: (1) cognitive principles have a strong influence on the decision making in Tangram (see Section 4) and (2) they are not all perceived and processed by humans in the same way and influence their decisions differently.

During Tangram solving, multiple cognitive principles might possibly influence different available actions. In order to abstract away from the single influence of one cognitive principle to one action, we introduce the concept of a unitary action choice, the action-option:

Action-Option. *An area of the silhouette defines an action-option when the edges limiting such area can be (partly) overlapped with the edges of a given piece. Such action-option is then characterized by 1. its location, 2. the matching piece type and 3. a strength value representing the influence of the respective cognitive principle(s)*.

Besides the first two features that are mostly involved in the identification of the action-option, the third feature, the strength value allows comparing among different action-options.

The application of action-options to the Tangram solving strategy is as follows: At each step, the action-option with the highest strength defines the location and the tan tan that best fits to this location, as most probably chosen by the player. This action is then expressed by the tuple (action-option, tan). If no action-option with fitting tan can be chosen, backtracking is necessary. In this case, from all previous actions, (action-option, tan), the tan that has the lowest frequency in training data will be chosen.

#### 5.1.2 State representation

In order to provide interfacing functions, the coordinator maintains an internal “virtual” representation of the current state by storing the list of the chosen (action-option, tan) still active (in the sense of actions taken and not backtracked) on the silhouette (i.e. the Tangram puzzle), together with the list of currently available action-options.

When the cognitive model responds with an action-option, the coordinator converts it into an actual action by creating the tuple (action-option, tan) and updating the state as a consequence. This is required by the presence of tans of multiple types: while in principle an action-option already includes the involved piece type, it is necessary to keep track of which individual piece is still free, in order to avoid unintentional backtracking or re-using of a placed tan.

#### 5.1.3 Updating actions

While the *cognitive model* module will provide the reasoning and eventually choose the action-option at each step, the processing and implementation into an actual action, (action-option, tan), is then delegated to the coordinator. Methods are thus present to implement both updating and backtracking functionalities.

### 5.2 Tangram application

The empirical study window was derived by an existing Tangram demo application and included interactive features which allowed the users to manipulate and move the tans. While such features were not required in the developed model, as no simulation of motor functions was planned, most of the data gathered during the empricial study was framed in the context of the application. As a result, the same window environment was maintained for better correspondence, and the model runs on an adaptation of the code for the original empirical study, providing just two main functionalities: it updates the window to represent the current state, and it captures the window screen so that it can be forwarded to the visual system for processing.

### 5.3 Visual system

An automated way to extract the action-option from the current puzzle's situation (or current context) from the empirical study window was required as hand-crafting the action-options for each available state was unfeasible due to the exponentially growing number of alternatives.

As a consequence, an external implementation based on classical computer vision techniques is proposed, its functionalities are limited by the following requirements in order to provide meaningful results: the algorithm should work on the silhouette edges, the algorithm should extract action-options that are plausibly exploited by humans, and the algorithm must associate an evaluation of the action-option's strength upon extraction.

The visual system was thus implemented in order to represent a structure cognitively inspired by the hierarchical structure of the human visual system. Given the strictly geometrical nature of the puzzle, and the fact that neurons in the primary visual cortex fire when matching certain oriented lines in the visual field (Hubel, 1982; Loffler, 2008), the algorithm simulates an hypothetical higher level construct able to identify certain line patterns representing the shapes.^3^ This was done via a pattern matching function applied on the edges of the current state and the template, using the sum of squared differences as similarity function.

Considering the binary nature of the images, a sum of absolute differences or a custom function might have been possible alternatives. However due to the amount of templates to match (for each shape and for each of its available rotations, which are discrete steps of 45°) the faster opencv (Bradski, 2000) implementation was preferred. Even though it is limited in the similarity functions options, it still provides acceptable results.

For each template, up to five candidate placements^4^ are extracted (parameter empirically chosen). These are then filtered for plausibility in two successive steps. First, it is checked whether the placement would intersect with other pieces currently placed, which can happen as the similarity function on edges might still tolerate limited intersection of corners. The second screening chooses only the placements that have some representation in the training data at the current phase of the solution.

**Figure 9:**
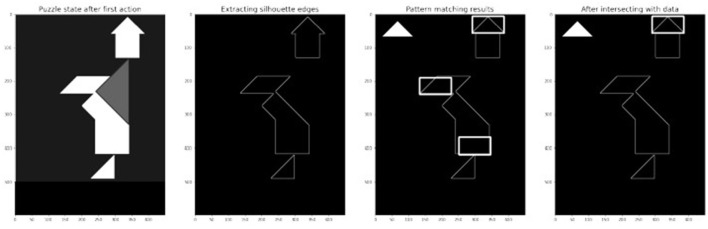
Steps of action-options extraction for small triangle at rotation 0.

**Figure 10:**
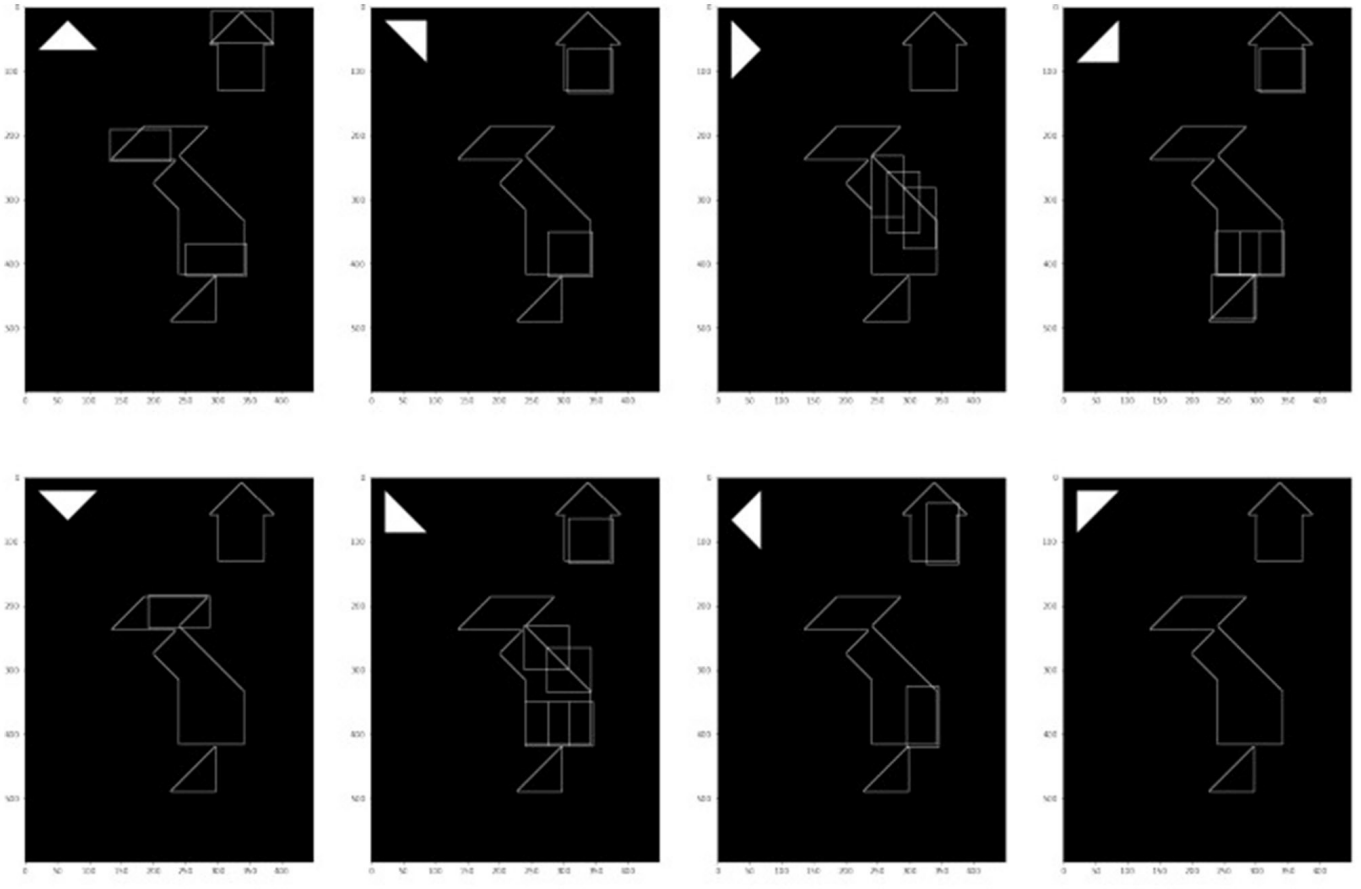
Results of the pattern matching for small triangles rotations, before intersecting with data.

The process is illustrated in Figure 9: the empirical study window returns the state of the puzzle and the image is binarized and its edges are extracted. For each possible rotation of each tan (here the small triangle at rotation 0 is shown, Figure 10 additionally shows the results for the other rotation angles), some potential matches are extracted. Finally the candidates are filtered for plausibility. The filtering also tackles an additional aspect: once an action-option is found its strength must be defined, which in turn will determine the baseline activation^5^ of the action-option in the model.

The following equation takes into account the strength of the extracted action-option, based on similarity scores and the available data:


(1) $$\begin{array}{l} strength_{i,j}=k_{d}*f_{i,j,phase}+k_{cv}*s_{i} \end{array}$$

where

**Table:**
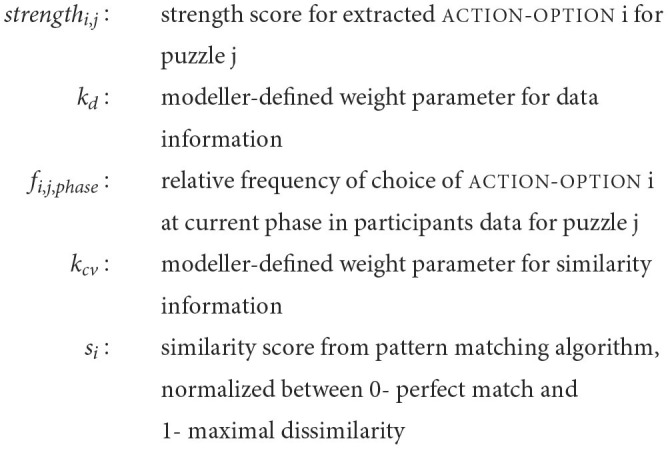


The values *k*_*d*_ and *k*_*cv*_ have been chosen empirically while performing the training. Motivated by the common assumption that the human's visual working memory is limited (Miller, 1956), only the six strongest action-options define the imaginal^6^ buffer to provide context and spreading activation.^7^ The number of action-options considered was chosen in order to be close to the 7 ± 2 from Miller's findings, but could probably be tuned as a parameter in further improvements of the framework.

It is thus possible to notice how the current implementation of the visual system has a particular focus on the aforementioned principle of Affordance (Section 4.1) coded as explicit pattern matching for the pieces. Nonetheless, frequency data from participants are also included in order to provide a proxy for the other principles.

Finally, the visual system attempts to recognize unfeasible regions (see Section 4.3, unfeasible region backtrack principle). In principle, such regions are parts of the silhouette in which no tan can be placed respecting the rules. At the current state, they cannot be matched directly as they come in various different shapes and sizes, but a property of the task can be exploited to identify some of them: the silhouette area must eventually be fully covered and all available pieces used. Considering this, if at any point in time no available placement is found for a piece, it means that its area is split between two or more separate regions that cannot accommodate it. In such cases, the presence of a problematic region is found and the coordinator will tag the next action as uncertain.

### 5.4 The cognitive model

The ACT-R model is tasked with choosing the next action to take at any given position, and it is mostly based on the baseline activation and spreading activation mechanisms. It mainly involves the goal module, the imaginal module, the declarative module and the procedural module.

**Figure 11:**
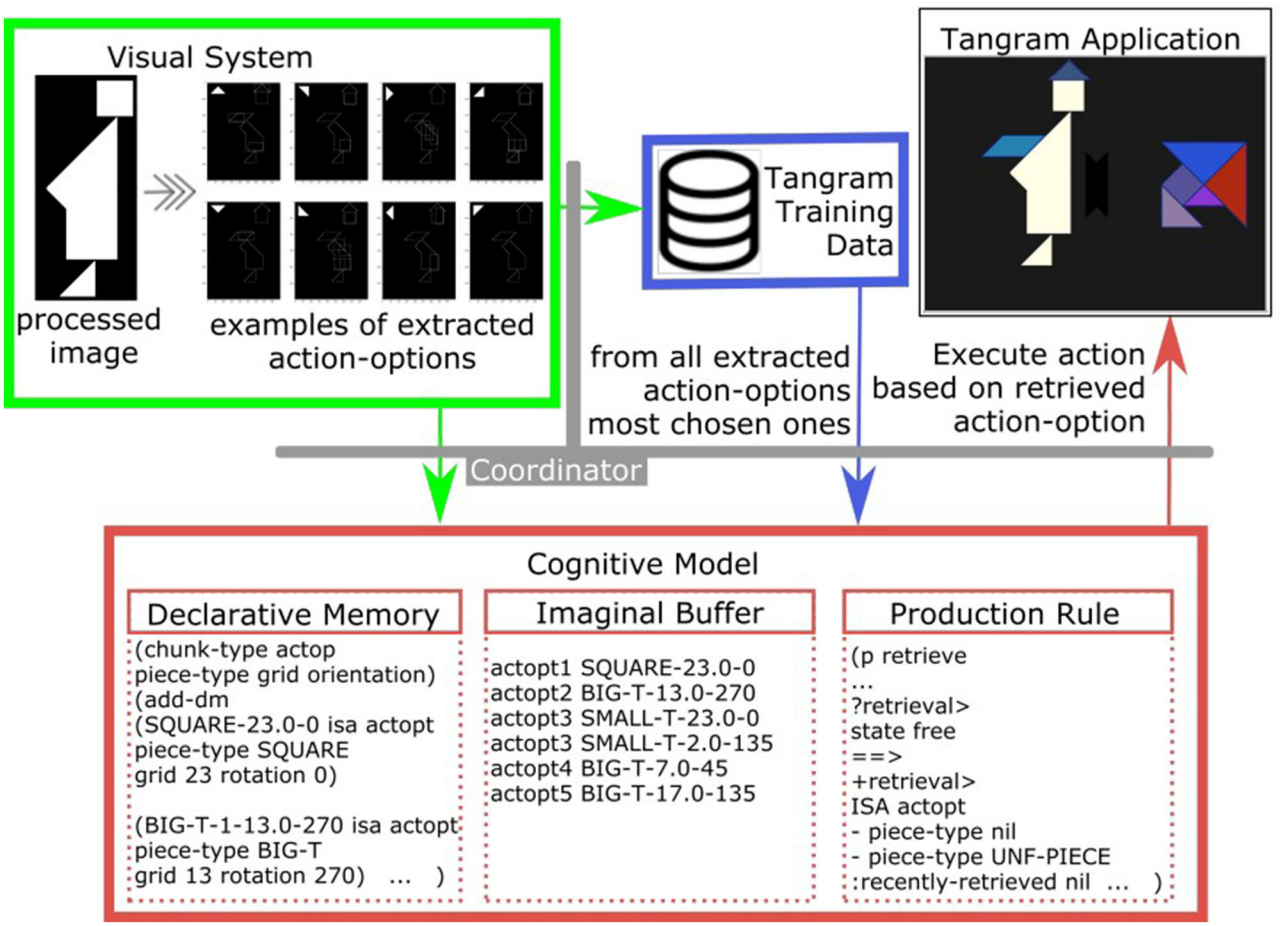
Example of action-options extraction in the visual system and their role in the model.

After the processing from the visual system the current available *action-options* are extracted (Figure 11, left), added to the declarative memory (Figure 11, bottom left) and the six strongest are loaded into the imaginal buffer (Figure 11, bottom middle). In this context, the imaginal buffer represents the “most noticeable” *action-options*, and helps their retrieval by the spreading activation mechanism through the respective production rule (as an example consider the production rule in Figure 11, bottom right). The main task of the module is to retrieve an action-option from the declarative memory and forward the decision to the coordinator.

Using the spreading activation mechanism, the action-options currently in context will have the highest chance of being retrieved. Retrieved action options can trigger either one of two different production rules. If it involves a piece, then the action-option will be validated and forwarded for the status update. The alternatives are that either a *unfeasible-region* chunk is retrieved, or that a retrieval error happens as there is no available action-option, or due to noise and low strength no action-option was strong enough to be retrieved.

These two events correspond to the two types of observed backtracking explained in Section 4.3 and respectively trigger unfeasible region backtrack and piece backtrack action lines.

The interaction between the two types of backtracking should be noted: in some cases, a given incorrect placement of a piece does not create any recognizable unfeasible-region, but will cause all the following actions to be problematic. This will not be noticed by any of the two individual productions, but it will eventually be possible to backtrack by their combination. Due to the constraint on *:recently-retrieved nil*,^8^ after various iterations of the **region_backtracking** with no solution, no new *action-options* will be available. At this point the **piece_backtracking** will be triggered, which will remove the weakest *action*, which has been stored separately when the action was taken. At later phases of the solution, the problematic action-options will be less frequent, as more participants will actually have solved the puzzle, and its strength in the model will thus be lowered.

It must be noted that simplifications are also implemented in order to deal with unfeasible region backtrack: as the model is currently not able to directly identify an unfeasible region, it instead relies on tagging chosen actions that become problematic. Thus the implementation of the strategy will differ from the suggested one by removing the first noticed action causing the problem and then proceeding in a queue-based manner until it is solved: a particular (production) rule will fire in the cognitive-model that will set the focus on solving the noticed issue until is not present anymore.

A slight simplification is also present with piece backtrack: it will generally work as described, backtracking the action related to the weakest chosen *action-option*, but its strength will be determined uniquely by the frequency data and not any geometrical representation.

## 6 Evaluation

Due to the relative novelty of the topic, there are no clear metrics or benchmarks on how to evaluate the Cognitive Tangram Solver performance on given human performance data. Generally it seems a challenging task to find good evaluation methods for cognitive models as they consider different aspects that are relevant (e.g. standard deviation, response times). In the case of the Cognitive Tangram Solver, the focus is to imitate the solving process applied by humans in this puzzle setting, including their backtracking strategies, in order to anticipate their next steps. However, the absolute order of steps is often not relevant. Taking as an example the {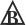, □, □} pattern in the solution of the House Tangram, it is possible to identify six possible sequences that can lead to the same pattern, any of which would be a desired behavior if shown by the model. A second challenge is that in the current implementation the model cannot distinguish between *imagining* and *doing* an action: a participant might realize while hovering over an area that the intended action will cause a problem region, and thus change the current placement (still considering as a single step). The Cognitive Tangram Solver does not implement “immediate thinking” and needs to actually conclude an action before possibly backtracking it. This is likely to cause longer solution procedures in case of mistakes.

In this section, we aim at providing the best possible picture of the behavior of the Cognitive Tangram Solver in relation to the human data. We first propose three variations of the CTS in Section 6.1. After that, Section 6.2 introduces four evaluation methods, including classical statistics and self-developed methods that focus on the solving process by humans, and provides the results with respect to the CTS variations.

### 6.1 Variations of the Cognitive Tangram Solver

The CTS framework includes a large number of parameters and design choices that can be optimized and discussed. Due to the exploratory nature of this work, and considering the additional need to identify acceptable metrics for evaluation, we decided to limit the analysis to few controllable and significant parameters. As a result, the CTS variations mostly differ on the relative weight of the parameters defining the strength of the action-options:

Vision-CTS The strength of an action-option is mainly defined by the template matching error function, applied by the template matching algorithm described in Section 5: the lower the error, the stronger the action-option. This implies a high influence from the best fit principle.
Frequency-CTS The strength of a action-option is mainly guided by the participants data. action-options are extracted via template matching as described in Section 5, but the training set is the main contributor to the successive validation. This approach implies a high influence from the other principles.
Balanced-CTS The strength of a action-option is derived both by the data and the template matching. The frequency of choice suffers a penalty depending on the size of the template matching error. This approach is a combination of vision-CTS and frequency-CTS.

### 6.2 Methods and results

The evaluation was done for the House and the Monk tangram. The first three evaluation methods give us insights on how closely the Cognitive Tangram Solver reproduces data similar to the ones in the empirical study. For each of these versions, CTS ran 30 times and the runs where aggregated. The activation noise specified in ACT-R caused the variations. We will briefly describe each method in more detail and then show their results.

#### 6.2.1 Overall statistics

The mean and standard deviation of the number of steps to reach the solution is calculated and compared to the participants' data to evaluate compatibility. This is expected to be most affected by the difference between “imagining” and “doing” an action. Additionally, the ratio of “perfect solutions” is compared: to bypass the issue with imperfect backtracking, it is evaluated how often the participants and the model manage to solve the puzzle in the minimal number of steps (7, one per tan).

Table 2 shows the overall statistics. In this and the following tables, the evaluation value with respect to the test set is in brackets next to the evaluation value with respect to the training set. Comparing the mean and standard deviation of the participants' data and the three models, all three models seem to lie within the range of the training and the test set values. The last column shows the perfect strategy ratio, which is the fraction of participants that solved the puzzle with the minimum amount of steps (seven). It seems that there is quite a discrepancy between the training and the test set for both Tangrams. Additionally, the percentages seem to suggest that none of the puzzles was significantly more difficult than the other: for the House Tangram, 43% of the participants applied a perfect strategy, but in the test set it was only 22%. In the case of the Monk Tangram, the discrepancy between the training and the test set is not as high. 27% of the participants in the training set applied a perfect strategy, whereas in the test set, the percentage is slightly lower with 22%. When considering both sets, on average 38% and 25% of the participants applied the perfect strategy for House and Monk, respectively. Interestingly, the balanced-CTS's performance is similar to the participants' performance for House tangram, which is not the case for the Monk tangram. For both tangrams, the vision-CTS performs the best regarding the perfect-strategy ratio but also diverges most from the participants' performance. A difference between the groups is noticeable despite them still being comparable. This suggests a consistent variability between different participants, and might reduce the informative value of these statistical results. Nonetheless, by considering the mean and standard deviation, it is still possible to infer some degrees of accordance with the performances of the models which still show a degree of alignment to the data sets, especially in the case of House Tangram. The ratio of perfect solutions appears instead to be widely ranging even among the participants, and might thus not offer useful insights in the evaluation.

**Table 2:** Overall statistics for total steps counts (test set values in brackets).

**Tangram**	**Type**	**Mean**	**Standard deviation**	**Perfect strategy ratio**
House	Training (test)	10.8 (8.9)	6.9 (2.3)	0.43 (0.22)
	Balanced-CTS	10.8	5.4	0.32
	Frequency-CTS	10.3	5.1	0.48
	Vision-CTS	10.2	5.5	0.58
Monk	Training (test)	12.4 (12.7)	6.5 (5.1)	0.27 (0.22)
	Balanced-CTS	13.1	6.5	0.42
	Frequency-CTS	11.4	5.7	0.35
	Vision-CTS	14.2	7.0	0.45

#### 6.2.2 Heatmaps comparison and choice error

During the analysis of data, heatmaps were produced which showed the relative frequency with which each tan type is chosen at each step. Even from a purely visual inspection, the heatmaps allow to consider similarities in the solution patterns. The data from model runs is thus similarly processed, and the results are compared. Following Schunn and Wallach (2005) we do not consider the $X^{2}$ or ANOVA, but use the root mean square error (RMSE) as the evaluation metric. The RMSE between the histograms at each step is calculated and averaged as follows:


(2) $$\begin{array}{l} {\text{RMSE}}_{s}=\sqrt{\overset{5}{\underset{p=1}{\sum }}\frac{{\left(h\left(p,s,\text{model}\right)-h\left(p,s,\text{data}\right)\right)}^{2}}{5}} \end{array}$$

where *s* is the step, *p* is the piece-type identifier, *h*_*s*_(*p*, model) is the frequency histogram value of piece-type *p* in the model, at step *s* and *h*_*s*_(*p*, data) is the frequency histogram value of piece-type *p* in the data, at step *s*.

**Figure 12:**
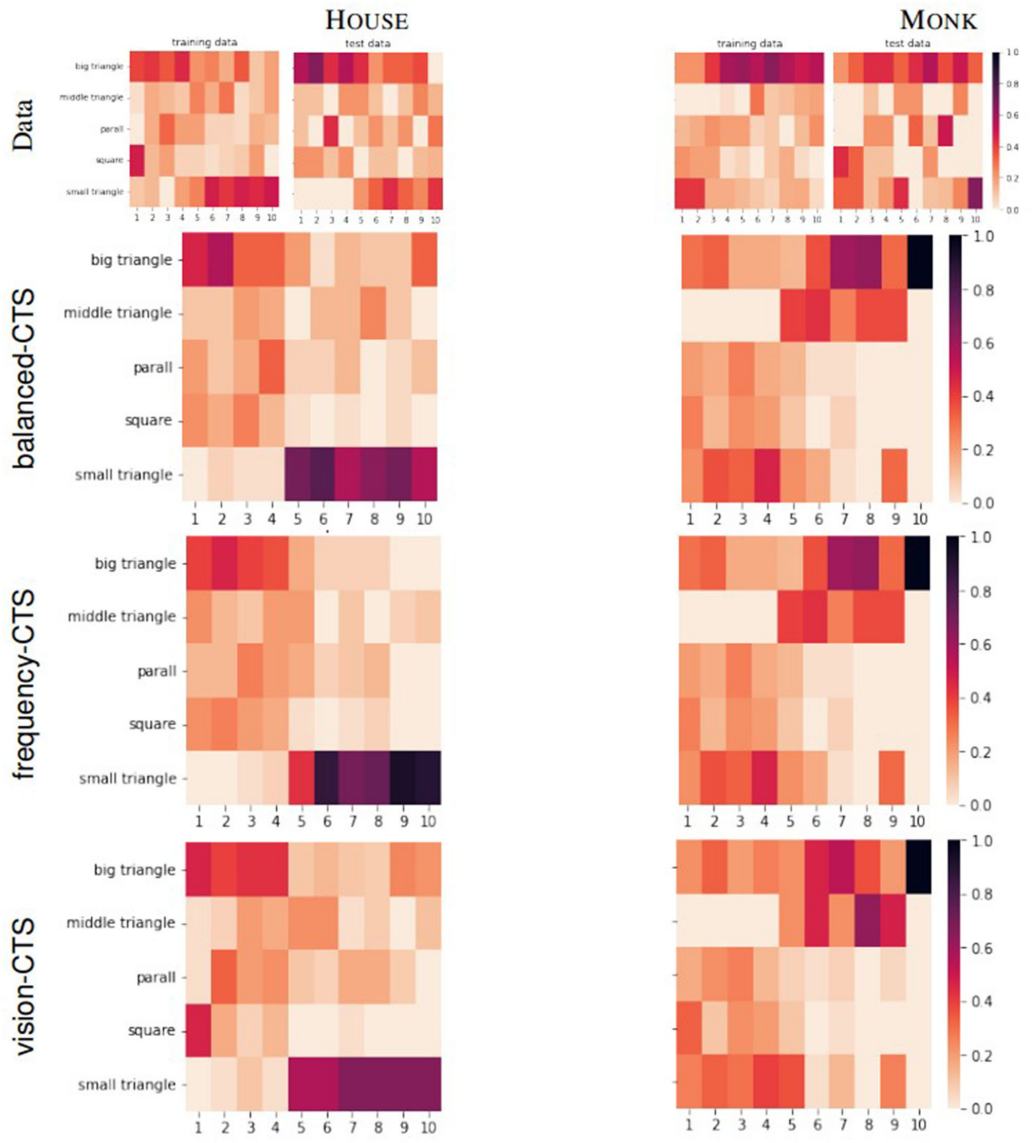
Heatmaps of the participants' data for House **(left)** and Monk **(right)** compared to the heatmaps produced by the three models.

The third column in Table 3 shows the heatmap RMSE and choice error. The heatmaps shown in Figure 12 give additional insights to their RMSE value. The horizontal axis denotes the step and the vertical axis denotes the tan that was chosen at this step. The darker the color of the bock, the higher the percentage of participants who have chosen the respective tan at that step. At first glance, the heatmaps of the data for both the House and Monk Tangram, are very similar to the heatmaps produced by the models. For the House tangram (heatmaps on the top left of Figure 12) a tendency to use the big triangles in the initial 1–4 steps and to use the small triangles in the last 5–10 steps can be observed in all heatmaps. The heatmap of the training data (left) shows lighter colors for these steps which indicates a more heterogenous group of the individuals. For the Monk tangram (four heatmaps in the bottom of Figure 12) a less strong tendency can be observed as the used tans among the different steps is more distributed.

**Table 3:** Heatmap RMSE, plausibility and prediction accuracy of the three CTS variations (test set values in brackets).

		**Heatmap RMSE**	**States plausibility**	**Prediction accuracy**
**Tangram**	**Type**	**training (test)**	**training (test)**	**training (test)**
House	balanced-CTS	0.14 (0.18)	0.46 (0.34)	0.50 (0.50)
	frequency-CTS	0.15 (0.19)	0.53 (0.42)	0.38 (0.43)
	vision-CTS	0.12 (0.17)	0.47 (0.38)	0.50 (0.35)
Monk	balanced-CTS	0.15 (0.18)	0.59 (0.19)	0.41 (0.30)
	frequency-CTS	0.16 (0.19)	0.59 (0.16)	0.39 (0.39)
	vision-CTS	0.16 (0.19)	0.57 (0.20)	0.46 (0.39)

The highest values are highlighted in gray.

The evaluation methods presented so far are widely used and helpful to compare the performance of different approaches. However, they do not give us any insight on whether CTS uses similar solving strategies as the humans, let alone how well CTS anticipates the humans' next steps. For this purpose, we developed two additional evaluation methods, the step-by-step states plausibility and the predictive accuracy. An elaborate discussion on their role will be given in Section 7.

#### 6.2.3 Step-by-step states plausibility

First, we consider the plausibility of how the puzzle state evolves with the model's actions. In this regard a state consists in the list of (grid_loc, rotation) values for each tan that is currently placed inside the solution grid. The steps from 3 to mean+1*sd (varying on the puzzle) are considered, trying to capture the majority of the available data. Each state in the model runs is compared with the user states at step ± offset, where offset is a flexibility parameter increasing with the number of steps, in order to exclude mismatches due to longer backtracking. The overall accuracy of the model is then computed by considering the states presenting matches with the data over the overall number of model states.

The fourth column in Table 3 shows the step-by-step states plausibility. The plausibility values reflect whether the states obtained by the model at a given step also appear among the users' states around the same step, trying to provide a performance scoring that captures the plausibility of the model's strategy. It is possible to notice how generally all the models perform worse for the House Tangram, and significantly worse for the Monk Tangram. Predictably, the frequency model has generally high training-set performances, but it is interesting to notice how the vision model has instead relatively higher performance in the test set, suggesting once more how an affordance based on shape similarity might be guiding the strategy.

#### 6.2.4 Prediction accuracy

This last proposed evaluation metric also considers contextual information, and compares the model's actions in the context of the game sequence. It defines an accuracy evaluation when the framework is used as a predictor, predicting the next step when any participant is solving the Tangrams. By minimal changes in the CTS, it is possible to have it predict the plausible next action at any given step during a participant's solution process. Trying to predict the exact following move would likely be too restrictive: consider as an example the roof pattern in the House tangram as shown in Figure 4 left. Once the big triangle is placed, at the current state it would be hard to distinguish the reasoning for which the square would be placed before the parallelogram, or vice versa. As a consequence, a prediction is accepted as valid if the suggested action happens in the following 2 steps.

The last column in Table 3 shows the prediction accuracy. The predictive accuracy evaluates the anticipatory performance of the model by running the trials of the participants and having the model predict the following move (accepting up to two steps forward as a match, due to the lower importance of strict sequences). Once more, different models perform differently in the two Tangrams, with the vision model having overall better training set accuracy, and with the frequency model improving in test set accuracy.

It is generally possible to notice a certain degree of overfitting, which can be expected when including the training data, which is still mostly limited to 5-10 percentage units. While the models' performances have a significant variation between the Tangrams, in this aspect the frequency-CTS seems more accurate.

#### 6.2.5 Results summary and discussion

Summarizing the observed results, no system shows consistently better results with respect to the others, neither within nor between the different puzzles. It is still possible to notice how the vision-CTS has the best performance in most cases, which could point toward a certain importance of the affordance principle.

It must be noted how the three models only focused on a limited number of variable parameters (specifically, the relations between data and similarity score for the strength in action-options), but a more comprehensive study could probably benefit from extending the scope to other evaluations too.

In any case, as the framework was meant to be considered a proof-of-concept for the application of the described cognitive principles, we suggest for these results to be seen as a basic benchmark, to be used and expanded upon for future attempts and studies.

## 7 Conclusion and future work

The intended contributions of the presented approach, which were presented in the introduction, can be summarized as: (a) an empirical study to an adequate problem solving geometrical task, (b) A novel hypothesis for human strategies for this task, and (c) A computational framework to reproduce the strategies and predict human behavior in the solution process. The first aspect is addressed by the design, the analysis and the results of the Tangram empirical study in Section 3. The second aspect to define hypotheses for human strategies, so-called cognitive principles, is addressed in Section 4. Finally, the third aspect is provided by the Cognitive Tangram Solver itself, which is a hybrid framework that integrates object recognition and cognitive modeling. This last aspect also includes an evaluating part, i.e. in how far the CTS is able to reproduce the strategies and predict human behavior in the solving process. This is addressed by the last two proposed evaluation methods in Section 6, the step-by-step stages plausibility and the prediction accuracy. Overall, even though CTS does not fully understand common mistakes, it can weakly anticipate the human's next steps: when CTS backtracks, we can assume that humans also has *difficulties* in finding a solution immediately.

Finally, as discussed in the introduction, the overall objective we are aiming at is the development of systems that can interpret the state of a task, in particular when there is no definite solution path. Even though the final goal is not known, the system needs to have a sense of anticipating difficulties and pitfalls, in order to predict individuals' traces of action and optimally support them whenever needed. As this objective contains a variety of highly demanding challenges, we have only addressed some of these challenges in a very controlled environment. The work presented offers a proof-of-concept showing the potentiality of the approach and justifies further studies in the field.

The presented approach can be improved in the future considering several aspects. It necessarily renounces some of the principles for practical implementation, a more fine-grained analysis that involves the strength definition should be considered.

In the specific case of this empirical study the available data were strictly dependent on the implementation of the empirical study window: the coordinates for the placements of the tans were expressed with respect to the empirical study window, and the tans themselves were defined as shapes within the framework. Even though most cognitive architectures provide a model of visual mechanisms, it seems that reproducing the setting purely within a architecture would likely have caused loss of precision in the shapes and coordinates definition, besides an additional overhead due to the lack of established methods for complex image manipulation. The development of visual recognition systems within cognitive architectures would be beneficial for the implementation of these types of tasks.

## Data availability statement

The datasets presented in this study can be found in online repositories. The names of the repository/repositories and accession number(s) can be found below: https://github.com/Thezamp/tangram-solver.

## Ethics statement

The requirement of ethical approval was waived by Ethics Committee of the Institute for Psychology and Ergonomics (IPA) at Technische Universität Berlin for the studies involving humans because Ethics Committee of the Institute for Psychology and Ergonomics (IPA) at Technische Universität Berlin. The studies were conducted in accordance with the local legislation and institutional requirements. The participants provided their written informed consent to participate in this study. Written informed consent was obtained from the individual(s) for the publication of any potentially identifiable images or data included in this article.

## Author contributions

GZ implemented the original model of the CTS and performed the extraction and analysis of the model results, defining together with ED the metrics to be used. ED provided support and supervision during the model development and results analysis, worked as main reviewer for the manuscript, and contributed to most of its structure. LH organized the human experiment and gathered the data, analyzed the demographics of participants, contributed in the review of the results, and additionally she curated the bibliographic aspects and consistency. NR wrote most of the introductory sections, was heavily involved in the literary review for the related works, and also worked as supervisor in the cognitive aspects of the model. All authors collaborated in the related work section based on their expertise topics. All authors contributed to the article and approved the submitted version.

## Footnotes

^1^In the following, screenshots showing tans or Tangram puzzles are taken from an adapted version of http://python4kids.net/downloads/py4k_cda4/turtledemo-python31/tdemo_games/tangram.py.

^2^Here, the correct placement of the triangle is defined by a discrete value in the 5 x 5 grid determined according to where the center point of its hypotenuse is located. For the big triangle this placement is shown in the Monk Tangram by the small black cross just above the horizontal line in location 17 in Figure 6. It must be noted that small pixel differences can influence grid values, thus introducing a noise component in the system.

^3^However, the intention was not to reproduce participants' visual behavior such as processing times but to provide a model with the ability to recognize shapes.

^4^Please notice that in the currently available version of the framework, such candidates are named “landmarks” and not “action-options”. This is due to a previous version of the framework which had a focus on the “technical” implementation, and will likely be changed in future revisions of the code.

^5^In the ACT-R architecture, this represents the likelihood that a given piece of information is extracted from the declarative memory when a retrieval process is triggered (i.e. when asking to retrieve a “geometrical shape”, retrieving a square might be more likely than retrieving an hexagon).

^6^ACT-R representation of information chunks related to the current task and context.

^7^ACT-R mechanism that allows contextual information to influence retrieval: chunks in the imaginal buffer related to context and task increase the activation of information chunks in the declarative memory beyond the baseline activation (Bothell, 2022, p.290ff).

^8^Parameter that will avoid the retrieval of the same action-option (and in general, informtion chunk) multiple times in a row.
